# Phytochemical Composition and Antioxidant and Anti-Inflammatory Activities of *Ligularia fischeri* Turcz: A Comparison between Leaf and Root Extracts

**DOI:** 10.3390/plants11213005

**Published:** 2022-11-07

**Authors:** Tae-Hyu Kim, Van-Long Truong, Woo-Sik Jeong

**Affiliations:** Food and Bio-industry Research Institute, School of Food Science & Biotechnology, College of Agriculture and Life Sciences, Kyungpook National University, Daegu 41566, Korea

**Keywords:** antioxidant, anti-inflammation, non-polar compounds, *Ligularia fischeri*

## Abstract

*Ligularia fischeri* Turcz leaves are widely consumed and have multiple health benefits. We aimed to evaluate the differences in the phytochemical composition and biological properties of the root and leaf extracts from *L. fischeri*. The root extract exhibited higher antioxidant capacity and total flavonoid levels than the leaf extract. GC/MS analysis revealed the presence of various volatiles, diterpenoids, sesquiterpenes, and other non-polar compounds. Moreover, these extracts enhanced cellular antioxidant defense by reducing the level of reactive oxygen species and upregulating the expression of catalase and heme oxygenase-1 in lipopolysaccharide (LPS)-stimulated RAW 264.7 cells. The root and leaf extracts also exerted anti-inflammatory effects by suppressing nitric oxide production and diminishing the levels of inducible nitric oxide synthase, cyclooxygenase-2, and interleukin-1β in LPS-stimulated macrophages. Overall, these findings suggest that *L. fischeri* root extract contains diverse bioactive compounds for the development of nutraceuticals or functional foods with antioxidant and anti-inflammatory activity.

## 1. Introduction

Aerobic organisms, including humans, use oxygen to synthesize or decompose substances through various oxidation-reduction reactions in the body to obtain energy and survive. Physiologically, these oxidation-reduction reactions must be balanced. However, redox homeostasis is sometimes disrupted by diverse internal and external factors, such as toxicants, immune reactions, ultraviolet radiation, and pollutants [1]. Cellular redox imbalance leads to the overproduction of reactive oxygen species (ROS) and other oxidants, resulting in oxidative stress that directly or indirectly causes damage to cellular macromolecules, such as lipids, proteins, and nucleic acids, shifting their normal functions [2]. Recent studies have demonstrated an interrelationship between oxidative stress and inflammation [3]. During acute or chronic inflammation, inflammatory and immune cells simultaneously produce ROS and reactive nitrogen species (RNS) and release pro-inflammatory enzymes and cytokines, such as inducible nitric oxide synthase (iNOS), cyclooxygenase-2 (COX-2), and interleukin (IL)-β, exacerbating pathological conditions [4]. Furthermore, ROS and RNS also enhance the pro-inflammatory response by upregulating inflammation-related signaling pathways [5]. Oxidative stress and inflammation are associated with the pathogenesis of various diseases, such as cardiovascular diseases, neurodegenerative diseases, inflammatory bowel diseases, aging, and cancer [6,7]. Fortunately, antioxidants, such as polyphenols and flavonoids act as reducing agents, metal chelators, and enzyme inhibitors and are capable of eliminating free radicals and inhibiting the formation of oxidants [8,9]. Therefore, dietary phytochemicals are essential for protecting against oxidative and inflammatory damage and preventing pathological conditions.

*Ligularia fischeri* Turcz (Gomchwi in Korean), belonging to the family Asteraceae, is an herbaceous perennial plant native to the alpine wetlands of East Asia. It is widely cultivated in greenhouses and shady forest fields in South Korea, China, and Japan. In Korea, young leaves are often harvested from April to May and used in salads, as pickled vegetables and spices owing to their bitter taste and rich flavor [10]. *L. fischeri* leaves have traditionally been used as a folk remedy to treat fever, back pain, rheumatoid arthritis, contusion, and hepatic diseases. The root of *L. fischeri* accounts for a considerable amount of the whole plant (approximately 40% of dried weight) and has been used in traditional Chinese medicine for the treatment of coughs, chronic bronchitis, and tuberculosis [11]. While *L. fischeri* has recently been cultivated to harvest leaves for food purposes, the pharmacological values of its root have attracted less attention. Recent studies have documented the antioxidant [12,13], anti-inflammatory [11,14], anti-cancer [15], neuroprotective [16], and hepatoprotective [17] activities of *L. fischeri*. The plant’s health benefits are attributed to the presence of abundant bioactive components, such as vitamins A, B1, B2, carotenes, niacin, phenolic compounds [17], flavonoids [18], sesquiterpenoids, and diterpenoids [19]. *L. fischeri* has higher vitamin A and β-carotene concentrations compared to other vegetables. In addition, *L. fischeri* is rich in aromatic and volatile oils, which produce distinctive flavors [20].

Previous studies on the phytochemical compounds of *L. fischeri* have mainly focused on the detection of phenolic compounds instead of the non-polar components of the whole extract. Most reports have focused on the leaves, and differences in the chemical composition and biological potential of the other parts have not been investigated fully. Therefore, we aimed to compare the non-polar compositions and biological activities of the leaf and root extracts of *L. fischeri*.

## 2. Results and Discussion

### 2.1. Total Phenolic and Flavonoid Contents and Chemical Composition of L. fischeri Leaf and Root Extracts

Phenolic compounds are abundant specialized metabolites of plants and an essential part of the human diet owing to their antioxidant activity and hepatoprotective, cardioprotective, anti-inflammatory, and anti-cancer effects [21]. Phenolic compounds can be used as natural preservatives against oxidative deterioration and bacterial contamination [22,23]. Phenolics have superior antioxidant capacity; thus, the phenolic content can be a major factor affecting the antioxidant capacity of plant-derived nutraceuticals and functional foods [21].

Table 1 presents the total phenolic and flavonoid levels of the ethanol extracts of *L. fischeri* leaves and roots. The total concentration of phenolic compounds in the leaf and root were 13.03 ± 0.11 mg GAE/g and 14.38 ± 0.21 mg GAE/g, respectively. No significant difference in the total phenolic content was observed between the leaves and roots. However, the total flavonoid content in the root extracts (2.45 ± 0.33 mg CE/g) was considerably higher than that in the leaf extracts (0.69 ± 0.22 mg CE/g). These results are inconsistent with those of a previous study reporting higher amounts of phenolics and flavonoids in the leaf extracts than in the root extracts [24]. These discrepant results may be due to differences in extraction methods and plant cultivation. Recent studies have indicated that caffeoylquinic acid (CQA) derivatives, such as 5-monoCQA, 3,4-diCQA, 3,5-diCQA, 4,5-diCQA, 1,3,4-triCQA, and 3,4,5-triCQA, are the major phenolic constituents that contribute to the antioxidant activity of *L. fischeri* extracts [16,25,26,27].

Additionally, *L. fischeri* contains considerable amounts of volatile and hydrophobic compounds, which may contribute to its health benefits. A previous study compared the protective abilities of *L. fischeri* root methanol extract and its fractions, including chloroform and ethyl acetate fractions, against ulcerative colitis in mice [11]. They indicated that the chloroform fraction was more effective than the methanol extract, whereas the ethyl acetate fraction elicited negligible therapeutic effects. These observations suggest that hydrophobic compounds in *L. fischeri* exert antioxidant and anti-inflammatory effects, thereby contributing to anti-colitis activity. In the present study, non-polar compounds in the roots and leaves of *L. fischeri* were characterized using GC/MS analysis and the chromatograms were illustrated in Figure S1.

We identified 56 and 68 compounds in the leaf and root extracts, respectively, of which only 13 were common between the two (Table 2). Principal component analysis (PCA) was used to emphasize variations and to visualize patterns within the dataset. Figure S2 shows the PCA plot with respect to principal component 1 (PC 1) and PC 2, accounting for 97.8% of the total variance in the dataset. More specifically, PC 1 accounted for 96.9% of the variance, while PC 2 was responsible for 0.9%. The score plot of PCA based on the profiles of non-polar compounds revealed a clear separation between the root and leaf groups. However, the precise reasons for this difference are unknown. Germacrene D (6.44%), β-maaliene (2.15%), 6,7-dimethoxy-3,4-dihydroisoquinoline (5.42%), neophytadiene (3.84%), cetene (3.51%), cyclocolorenone (2.37%), phytol (11.51%), 1-Monolinolein (4.72%), 2-Monolinolenin (4.04%), stigmasterol (5.89%), and γ-sitosterol (6.10%) accounted for approximately 55.99% of the total hydrophobic compounds in the leaf extracts. The root extract was rich in 2-[(1aS,4aS,7R)-4a-methyldecahydrocyclopropa[d]naphthalen-7-yl]-2-propanol (2.78%), 1,2,5-Trimethylpyrrole (10.67%), (E)-3-(1-Phenylprop-1′-en-2′-yl))-pentane-2,4-dione (2.65%), 4-methylcyclohex-3-enecarbaldehyde (6.16%), 3,4-dihydro-2-(methoxymethyl)-4,4-dimethyl-5-phenyl-2H-pyran (30.03%), and 12-methoxy-18-norpodocarpa-8,11,13-trien-19-ol (5.05%), accounting for 57.34% of the total non-polar compounds. Consistent with this study, a previous study showed the presence of volatile compounds in *L. fischeri*, such as terpinolene, caryophyllene, humulene, eremophilene, farnesene, and hexadecanol that exert anti-inflammatory activity mainly by inhibiting the production of inflammatory factors [20]. Several compounds, such as methyl linoleate, linoleic acid, methyl linolenate, and γ-sitosterol might potentially be the effective components of the chloroform fraction of *L. fischeri* root methanol extract and contribute to its anti-colitic activity [11]. Previous reports have demonstrated the presence of sesquiterpenes and diterpenoids in *L. fischeri*; however, their therapeutic potential remains unknown [19,28,29].

Overall, these findings suggest that *L. fischeri* contains a large number of phytochemicals, including aromatic and volatile constituents, hydrophobic compounds, phenolics, and flavonoids, which have various health benefits.

### 2.2. In Vitro Antioxidant Activities of L. fischeri Leaf and Root Extracts

Chemical-based antioxidant activity assays are utilized to assess the antioxidant potential of natural substances, foods, or dietary supplements, providing preliminary evidence of the antioxidant capacities of a plethora of extracts and phytochemicals. However, a single assay cannot represent the total antioxidant activity of different plant-derived extracts because of their varying chemical composition and phytochemical nature [30]. Therefore, five different assays (DPPH, ABTS^+^, NO radical scavenging, FRAP, and TAC) were employed to evaluate the antioxidant activity of *L. fischeri* leaf and root extracts.

**Figure 1:**
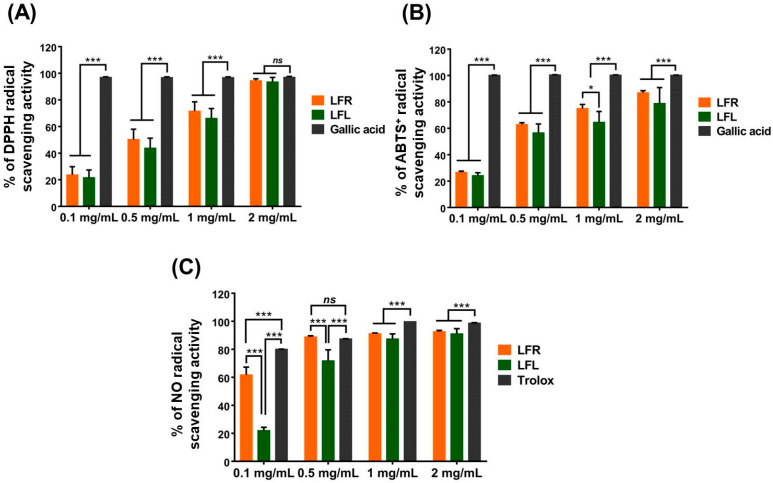
Antioxidant activities of root and leaf extracts from *L. fischeri* in a cell-free system. (**A**) DPPH, (**B**) ABTS^+^, and (**C**) NO radical scavenging capacity. Results are presented as the mean ± S.D. of at least three independent experiments. * *p* < 0.05 and *** *p* < 0.001 are considered statistically significant differences. LFR and LFL: *L. fischeri* root and leaf extracts, respectively.

DPPH and ABTS^+^, which are stable organic radicals, are utilized to investigate the antioxidant capacities of natural compounds. Both assays are based on reduction via single-electron transfer or quenching via hydrogen atom transfer by an antioxidant [31]. As shown in Figure 1A,B, *L. fischeri* root and leaf extracts were able to strongly scavenge DPPH and ABTS^+^. The DPPH scavenging activities of root and leaf extracts at 2 mg/mL were 94.4 ± 1.37% and 93.4 ± 3.45%, respectively, which was identical to that of gallic acid (97.0 ± 0.65%) at the same concentration, while the ABTS^+^ scavenging activities were 86.9 ± 1.56% and 78.8 ± 12.06%, respectively, which was lower than gallic acid (100 ± 0.12%). No difference in the radical scavenging potential was observed between the leaf and root extracts.

Nitric oxide (NO) is an abundant species with a half-life of a few seconds in aqueous environments. NO can rapidly diffuse through the cytoplasm and plasma membrane and is involved in various physiological and pathological processes. Under oxidative bursts caused by inflammatory conditions, NO may react with superoxide anion radicals to generate a highly active oxidant, peroxynitrite anion, which can destroy DNA and lipids [32]. In living organisms, NO overproduction results in nitrosative stress, a deleterious process. In the present study, both root and leaf extracts showed excellent NO scavenging abilities, as evidenced by percent inhibition at 2 mg/mL of 93.8 ± 2.37% and 94.8 ± 4.46%, respectively, comparable to Trolox (98.7 ± 0.35%) at the same concentration (Figure 1C). Interestingly, at lower concentrations (0.1 and 0.5 mg/mL), the NO-scavenging capacity of the root extract was more pronounced than that of the leaf extract.

The FRAP assay can be used to examine the reducing power of extracts, fractions, or single compounds and is based on the reduction of a ferric salt (Fe^3+^) to ferrous salt (Fe^2+^) by an electron transfer reaction. Table 1 showed that the reducing power of the root extract was 20.47 ± 1.59 mg GAE/g, which was remarkably higher than that of the leaf extract (10.07 ± 2.30 mg GAE/g). Similarly, the phosphomolybdenum method, based on the reduction of Mo (VI) to Mo (V) in the presence of antioxidants, can also measure the total antioxidant capacity of extracts, fractions, or single compounds. In this study, the total antioxidant capacity of the root extract (203.39 ± 8.11 mg TE/g) was significantly stronger than that of the leaf extract (101.60 ± 13.72 mg TE/g) (Table 1).

### 2.3. Antioxidant Activities of L. fischeri Leaf and Root Extracts in LPS-Stimulated RAW 264.7 Cells

Although many chemical-based antioxidant assays have been widely applied to examine and provide preliminary data on the antioxidant activities of natural products, foods, and dietary supplements, little is known about their effects in vivo under physiological temperature, humidity, and pH, as well as their mechanism of absorption, metabolism, and excretion [33]. Cell-based antioxidant assays help address these issues. Thus, this study further investigated the antioxidant capacities of the root and leaf extracts of *L. fischeri* using lipopolysaccharide (LPS)-treated RAW 264.7 cells.

**Figure 2:**
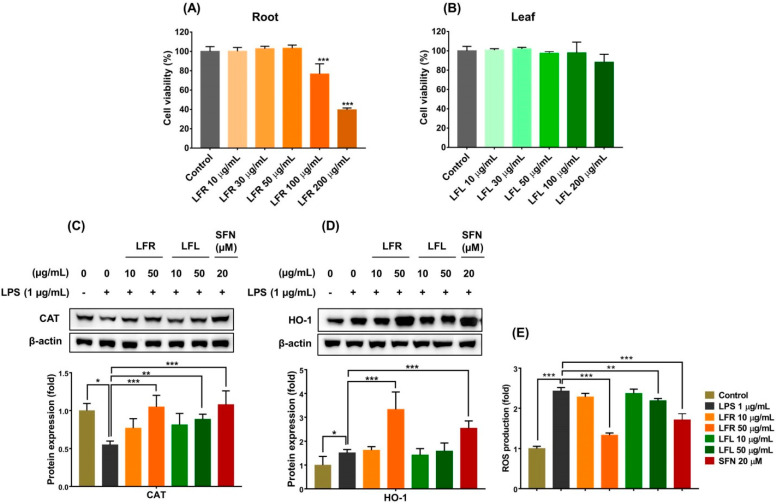
Cytotoxic effects and cell-based antioxidant activities of root and leaf extracts from *L. fischeri*. RAW 264.7 cells were incubated with various concentrations (10–200 µg/mL) of root or leaf extracts for 24 h. The cell viability was measured using MTT assay. (**A**,**B**) Cell viability for root and leaf extracts, respectively. The RAW 264.7 cells were pretreated with LFR or LFL for 1 h prior to LPS (1 µg/mL) incubation for an additional 12 h. (**C**) CAT expression. (**D**) HO-1 expression. (**E**) Intracellular ROS accumulation. Results are presented as the mean ± S.D. of at least three independent experiments. * *p* < 0.05, ** *p* < 0.01, and *** *p* < 0.001 are considered statistically significant differences. LFR and LFL: *L. fischeri* root and leaf extracts, respectively; SFN: sulforaphane; LPS: lipopolysaccharide.

First, the cytotoxic effects of LFR and LFL on RAW 264.7 cells were evaluated using MTT assay. The viability of RAW 264.7 cells was reduced by LFR at high concentrations (>100 µg/mL), while LFL up to 200 µg/mL did not affect cell survival (Figure 2A,B). Both LFR and LFL at 50 µg/mL were unlikely to cause significant cell death, and therefore, LFR and LFR concentrations <50 µg/mL were selected for further experiments.

In living organisms, cells protect themselves against oxidative stress by directly removing oxidants before they can attack vital cellular molecules and/or indirectly enhancing antioxidant defense systems, including antioxidant enzymes [34]. Endogenous antioxidant enzymes, including catalase (CAT), glutathione peroxidase, and superoxide dismutase, provide primary defense against massive oxidative assault by catalyzing various biological reactions to decompose and nullify ROS/oxidants [35]. Consequently, enhancing primary antioxidant enzymes could be a useful strategy for prevention of undesired pathological conditions. Biological efficacy expected from the consumption of food would be a prevention effect rather than a therapeutic effect. Thus, pretreatment seems to be more reasonable than post-treatment. In this study, RAW 264.7 cells were pretreated with extracts for 1 h prior to LPS challenge. The results showed that LPS (1 µg/mL) treatment considerably decreased the CAT expression that was significantly restored by pretreatment with the root or leaf extracts (Figure 2C).

In addition to primary antioxidant enzymes, heme oxygenase-1 (HO-1), a phase II antioxidant enzyme, plays a vital role in the maintenance of redox homeostasis. It catalyzes the degradation of heme to biliverdin, ferrous iron, and carbon monoxide, which exert cytoprotective activities [36]. Biliverdin is subsequently converted into bilirubin, which has a potent antioxidant with radical scavenging activity, by the action of biliverdin reductase. Moreover, bilirubin can also reduce ROS formation via inhibiting the activation of NADPH oxidase. Carbon monoxide generated by HO-1 acts as a gasotransmitter that has anti-inflammatory, anti-apoptotic and vasodilator properties via signaling transduction pathways. Free iron released from heme is neutralized by several cellular iron storage and transport pathways, induced by iron itself. These are composed of the activation of an iron efflux pump and simultaneous induction of ferritin, a major iron sequestering pathway. In addition, the action of HO-1 results in a decrease in intracellular heme content, limiting iron availability for Fenton’s reaction, preventing the assembly and activation of NADPH oxidase, and thereby reducing ROS generation [37].

Thus, to further investigate the antioxidant abilities of *L. fischeri* root and leaf extracts, the expression of HO-1 was examined. As shown in Figure 2D, the root extract remarkably upregulated the expression of HO-1, whereas the leaf extract did not affect the level of this enzyme in LPS-stimulated macrophages. The inductive effect of the root extract was better than that of sulforaphane, a well-known HO-1 inducer. Consequently, LPS-induced intracellular ROS accumulation significantly reduced in the presence of the root and leaf extracts, and the root extract appeared to be more effective than the leaf extract (Figure 2E).

Overall, these findings suggest that *L. fischeri*, especially the root portion, exerts cellular antioxidant effects by inducing primary and phase II antioxidant enzymes and scavenging free radicals.

### 2.4. Anti-Inflammatory Activities of Root and Leaf Extracts from L. fischeri in LPS-Treated RAW 264.7 Cells

Inflammation is a tightly regulated process involving pro-inflammatory and anti-inflammatory components that cooperatively act to eliminate pathogens and injured cells, prevent the spread of harmful agents to surrounding tissues, and restore tissue structure. Failure to regulate this process can lead to acute/chronic inflammation and pathogenesis of various diseases, such as arthritis, atherosclerosis, and inflammatory bowel diseases [38].

Macrophages contribute importantly to the innate immune system—the first line of defense against endogenous and exogenous pathogens. In response to inflammatory stimuli, such as endotoxins (e.g., LPS), inflammatory cytokines (e.g., tumor necrosis factor-alpha), and ROS/RNS, macrophages secrete a large number of growth factors and pro-inflammatory mediators (NO and prostaglandins), adhesion molecules, and chemokines along with pro-inflammatory cytokines (IL-1β and IL-6). The inducible enzymes, including iNOS and COX-2, are responsible for the generation of NO and prostaglandins at inflammatory sites, respectively, resulting in oxidative/nitrosative stresses that can cause complications, such as septic shock and various inflammatory conditions [39,40]. Inflammation is managed by regulating the release of pro-inflammatory mediators and cytokines, preventing inflammation-related diseases. Therefore, this study examined the anti-inflammatory activities of *L. fischeri* root and leaf extracts using LPS-stimulated RAW 264.7 cells.

**Figure 3:**
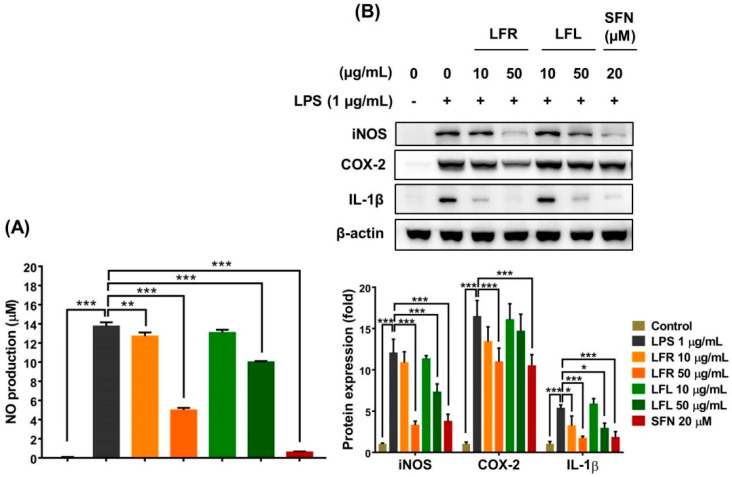
Anti-inflammatory activities of root and leaf extracts from *L. fischeri* in LPS-stimulated RAW 264.7 cells. The cells were pretreated with LFR or LFL for 1 h prior to LPS (1 µg/mL) incubation for an additional 12 h. (**A**) NO production. (**B**) iNOS, COX-2, and IL-1β expression. Results are presented as the mean ± S.D. of at least three independent experiments. * *p* < 0.05, ** *p* < 0.01, and *** *p* < 0.001 are considered statistically significant differences. LFR and LFL: *L. fischeri* root and leaf extracts, respectively; SFN: sulforaphane; LPS: lipopolysaccharide.

The results showed that LPS (1 µg/mL) considerably increased NO production in macrophages; however, pretreatment with root or leaf extract prevented this effect (Figure 3A). Additionally, the LPS-induced expression of iNOS and IL-1β was remarkably attenuated by the presence of root and leaf extracts in RAW 264.7 cells (Figure 3B). More importantly, the root extract reduced the COX-2 level, whereas the leaf extract did not show this effect. The inhibitory efficacy of the root extract on the inflammatory response was greater than that of the leaf extract. Overall, these data suggest that *L. fischeri* extracts exert anti-inflammatory effects by regulating the generation of pro-inflammatory factors and antioxidant enzymes and scavenging free radicals.

## 3. Materials and Methods

### 3.1. Materials

Lipopolysaccharide (Escherichia coli O127:B8), 2,2ʹ-diphenyl-1-picrylhydrazyl radical (DPPH), 2,2′-azino-bis-3-ethylbenzothiazoline-6-sulfonic acid (ABTS), Folin-Ciocalteu’s phenol reagent, 2,4,6-tris(2-pyridyl)-1,3,5-triazine (TPTZ), sodium nitroprusside (SNP), gallic acid, catechin, and Trolox were purchased from Sigma-Aldrich (St. Louis, MO, USA). All the antibodies were obtained from Santa Cruz Biotech (Santa Cruz, CA, USA) or Cell Signaling Technology (Danvers, MA, USA). All the other reagents used in this study were of the highest analytical grade.

### 3.2. Preparation of Ethanol Extracts of L. fischeri

*L. fischeri* was collected from a farm in Hongcheon (Gangwon-do, Korea) in August 2021. After discarding the damaged parts, the plants were washed and categorized into leaf and root parts. The 5 kg samples were air-dried at 55 °C and ground into fine particles. Fifty grams of dried powders of *L. fischeri* leaves and roots were extracted separately with 95% ethanol (1:10, *w*/*v*) for 24 h. The suspensions were filtered using Whatman filter paper Grade 2 (GE Healthcare, Chicago, IL, USA), and the residues were re-extracted by adding 95% ethanol. The filtrates were combined and concentrated using a rotary evaporator (Eyela, Tokyo, Japan) to obtain the *L. fischeri* leaf and root extracts (LFL and LFR, respectiveity).

### 3.3. GC-MS Analysis

The root and leaf extracts from *L. fischeri* were dissolved in hexane and subjected to GC-MS analysis using Agilent 7890A GC and 5975C MSD instruments (Agilent, Santa Clara, CA, USA). The nonpolar compounds were separated on a J&W DB-5ms GC column (60 m × 0.25 mm × 0.25 µm). Helium was used as the carrier gas, with a column-head pressure of 16.909 psi. The oven temperature was initiated at 50 °C for 5 min, programmed at 300 °C at a rate of 5 °C/min, maintained for 30 min, increased to 310 °C at a rate of 10 °C/min, and held for 10 min. The flow rate was set at 1 mL/min. A full scan mode (*m*/*z* 30–500) was used to detect all the target compounds. The compounds were tentatively detected by comparing the mass spectra of the peaks with those in the NIST library, and the percentage of each compound was calculated by normalizing the methods of at least three experiments.

### 3.4. In Vitro Colorimetric Assays

The antioxidant activities of the extracts were performed according to methodologies described in previous reports [41,42,43,44]. First, the total antioxidant capacity of the samples was estimated using the phosphomolybdenum method. Reaction mixtures, including 28 mM sodium phosphate, 0.6 M sulfuric acid, 4 mM ammonium molybdate, and tested samples, were heated at 95 °C for 90 min. DMSO was used as a blank control. After cooling, absorbance was measured at 695 nm. The total antioxidant capacity was calculated using a Trolox standard curve and displayed as an equivalent of Trolox (mg TE/g of dried extract).

For 1,1-diphenyl-2-picrylhydrazyl (DPPH) radical scavenging activity, various concentrations of samples were added to a 0.2 mM DPPH solution and left to stand for 30 min in the dark. DMSO was used as the blank control, and gallic acid was utilized as a positive control. Absorbance was recorded at 517 nm using a microplate reader (BioTek, Winooski, VT, USA). DPPH radical scavenging capacity was calculated as follows:

Percentage (%) of DPPH radical scavenging activity = [A_0_ − A_1_/A_0_] × 100

where A_0_ and A_1_ are the absorbances of the control and samples, respectively.

For ABTS^+^ radical scavenging activity, ABTS^+^ radicals were generated by mixing 7 mM ABTS solution with 2.45 mM potassium persulfate and allowing the mixture to incubate for approximately 12–16 h in the dark before use. After dilution with ethanol to obtain an absorbance of 0.700 at 734 nm, the ABTS solution was mixed with various concentrations of the samples. Absorbance was measured at 734 nm after incubation for 5 min. DMSO was used as the blank control, and gallic acid was utilized as a positive control. ABTS^+^ radical scavenging activity was calculated using the following formula:

Percentage (%) of ABTS^+^ radical scavenging activity = [A_0_ − A_1_/A_0_] × 100

where A_0_ and A_1_ are the absorbances of the control and tested samples, respectively.

For nitric oxide (NO) radical scavenging activity, the reaction mixtures, including 10 mM SNP in phosphate-buffered saline (PBS, pH 7.4) and different concentrations of samples, were incubated at room temperature for 150 min. The levels of the generated NO radicals were measured using Griess reagent (1% *w*/*v* sulfanilamide in 5% *v*/*v* phosphoric acid and 0.1% naphthyl ethylenediamine hydrochloride). DMSO was used as a blank control, and Trolox was used as a positive control. The percentage inhibition of the absorbance at 564 nm was calculated using the following formula:

Percentage of NO radical scavenging activity = [(A_0_ − A_1_)/A_0_] × 100

where A_0_ and A_1_ are the absorbances of the control and tested samples, respectively.

For ferric reducing antioxidant power (FRAP), the samples were mixed with a premixed cocktail solution (300 mM acetate buffer (pH 3.6), 20 mM ferric chloride, and 10 mM TPTZ in a ratio of 10:1:1). Absorbance was measured at 590 nm after 30 min of incubation at room temperature in the dark. DMSO was used as a blank control. The FRAP value was calculated using a gallic acid standard curve and expressed as an equivalent of gallic acid (mg GAE/g of dried extract).

### 3.5. Total Phenolic and Flavonoid Contents

Total phenolic content was measured using Folin–Ciocalteu phenol reagent according to a previously described method with slight modifications [45]. Briefly, the samples were vigorously mixed with Folin–Ciocalteu reagent for 5 min, followed by the addition of 20% (*w*/*v*) sodium carbonate. After a 60-min incubation at room temperature, the absorbance was recorded at 700 nm using a microplate reader (BioTek). The total phenolic content was calculated using a gallic acid standard curve and expressed as an equivalent of gallic acid (mg GAE/g of dried extract).

The total flavonoid content was measured using an aluminum colorimetric assay, as described previously [46]. The samples were mixed with 50% (*v*/*v*) ethanol and 5% (*w*/*v*) NaNO_2_ and allowed to stand for 6 min at room temperature. An Al(NO_3_)_3_ solution (10%, *w*/*v*) was then added to the mixture and incubated for 6 min. The reactions were stopped by adding 1N NaOH solution. Absorbance was recorded at 510 nm using a microplate reader (BioTek). The total flavonoid content was calculated using a catechin standard curve and expressed as an equivalent of catechin (mg CE/g of dried extract).

### 3.6. Cell Culture

The murine RAW 264.7 macrophage cells, acquired from American Type Culture Collection (Manassas, VA, USA), were maintained in Dulbecco’s modified Eagle’s medium supplemented with 10% fetal bovine serum, penicillin (100 U/mL), and streptomycin (100 µg/mL) in a humidified incubator at 37 °C with 5% CO_2_. The medium was replaced every two days.

### 3.7. Cell Viability

The cytotoxic effects of LFR and LFL on RAW 264.7 cells were determined using 3-(4,5-dimethylthiazol-2-yl)-2,5-diphenyltetrazolium bromide (MTT) assay. Briefly, the cells were seeded in 96-well plates and treated with LFR or LFL (10–200 µg/mL) for 24 h and followed by incubation with MTT reagent (5 mg/mL) for 3 h at 37 °C with 5% CO_2_. Formazan crystal was then solubilized in dimethyl sulfoxide and the absorbance was measured at 570 nm using a microplate reader (BioTek).

### 3.8. NO Production

The cells were seeded in 24-well plates and pretreated with LFR or LFL for 1 h prior to LPS (1 µg/mL) challenge for an additional 12 h. The NO concentrations in the culture media were measured using the Griess reaction. Briefly, the cell culture media were mixed with an equal volume of Griess reagent (1% sulfanilamide (*w*/*v*) in 5% phosphoric acid (*v*/*v*) and 0.1% naphthyl ethylenediamine hydrochloride (*w*/*v*)) and incubated at room temperature for 10 min. Absorbance was recorded at 550 nm using a microplate reader (BioTek).

### 3.9. Intracellular ROS Accumulation

The cells were seeded in 96-well plates and pretreated with LFR or LFL for 1 h prior to LPS (1 µg/mL) challenge for an additional 6 h. Intracellular ROS formation was detected using the cell-permeant reagent 2′,7′-dichlorofluorescein diacetate (DCFH-DA). Briefly, after treatment, the cells were washed twice with phosphate-buffered saline and incubated with 20 µM DCFH-DA for 1 h at 37 °C in the dark. After washing out the excess probe with PBS, fluorescence was read at the excitation of 485/20 nm and the emission of 528/20 nm by a microplate reader (BioTek).

### 3.10. Western Blot Analysis

The cells were seeded in 6-well plates and pretreated with LFR or LFL for 1 h prior to LPS (1 µg/mL) challenge for an additional 6 h. After being washed twice with ice-cold phosphate-buffered saline, the cells were homogenized in RIPA buffer (Cell Signaling Technology), and the protein content was measured using a Pierce^TM^ BCA protein assay kit (Thermo Fisher Scientific, Waltham, MA, USA). Equal amounts of protein were separated using SDS-PAGE gels and then transferred onto a polyvinylidene fluoride membrane using a semidry transfer system (Bio-Rad, Hercules, CA, USA). After blocking with 5% non-fat skim milk for 2 h at room temperature, the membranes were incubated with specific primary antibodies overnight at 4 °C and followed by hybridization with appropriate secondary antibodies for 3 h at 4 °C. Protein bands were detected using western blotting luminol reagent (Santa Cruz Biotechnology, Dallas, TX, USA).

### 3.11. Statistical Analysis

Data are presented as the mean ± standard deviation (S.D.) of at least three independent experiments. Statistical analysis was carried out using analysis of variance followed by Tukey’s multiple test (Prism Graphpad version 7.0, San Diego, CA, USA). *p* values < 0.05 were considered statistically significant.

## 4. Conclusions

The findings obtained from this study demonstrate that *L. fischeri* extracts are potential sources of phytochemicals, such as phenols, flavonoids, aromatic compounds, volatiles, and various hydrophobic compounds. The ethanol extract of the *L. fischeri* root, rather than the leaf, possesses antioxidant and anti-inflammatory activities due to its rich phytochemical content. These findings suggest that *L. fischeri* root extracts are a potent agent for the development of nutraceuticals and functional foods with antioxidant and anti-inflammatory properties.

## Figures and Tables

**Table 1:** Total phenolic and flavonoid contents and antioxidant capacities of root and leaf extracts from *L. fischeri*.

	Root Extract	Leaf Extract
TPC (mg GAE/g)	14.38 ± 0.21	13.03 ± 0.11
TFC (mg CE/g)	2.45 ± 0.33 ***	0.69 ± 0.22
FRAP (mg GAE/g)	20.47 ± 1.59 ***	10.07 ± 2.30
TAC (mg TE/g)	203.39 ± 8.11 ***	101.60 ± 13.72

Results are presented as the mean ± S.D. of at least three independent experiments. *** *p* < 0.001 values are considered statistically significant by the Student *t*-test. TPC: total phenolic content; TFC: total flavonoid content; FRAP: ferric reducing antioxidant power; TAC: total antioxidant capacity.

**Table 2:** Chemical composition of *L. fischeri* root and leaf extracts analyzed by GC-MS.

R.T (min)	Compounds	Leaf Extract (%)	Root Extract (%)
10.626	2,3-Butanediol	1.69 ± 0.04	-
16.850	Diethylacetic acid	1.64 ± 0.07	-
20.621	Butyl isobutyrate	0.67 ± 0.09	-
21.089	Hexyl isobutyrate	0.39 ± 0.04	-
21.478	Terpinolene	-	0.05 ± 0.01
24.727	p-Cymen-8-ol	0.19 ± 0.02	0.08 ± 0.01
26.385	Linalyl acetate	-	0.12 ± 0.01
27.542	1-Methylbicyclo[4.1.0]-heptane	0.06 ± 0.01	-
27.633	Isobornyl acetate	-	0.03 ± 0.01
27.939	Trans-pinocarvyl acetate	-	0.05 ± 0.001
29.11	Isojasmone	-	0.03 ± 0.02
29.323	Terpinyl acetate	-	0.14 ± 0.01
29.746	Alpha-Longipinene	-	0.01 ± 0.01
30.336	Copaene	0.12 ± 0.02	-
30.382	1-Tetradecene	-	0.03 ± 0.01
30.612	Calarene	0.14 ± 0.04	-
30.619	Beta-Elemene	-	0.23 ± 0.01
31.164	Aromadendrene	-	0.03 ± 0.01
31.247	Cyperene	-	0.04 ± 0.001
31.506	1-Methoxy-1,3-cyclohexadiene	0.25 ± 0.01	-
31.612	Caryophyllene	1.48 ± 0.28	0.11 ± 0.01
31.709	Dihydro-beta-ionone	-	0.03 ± 0.01
31.863	Alpha-Guaiene	-	0.02 ± 0.02
32.006	Cis- Beta-Farnesene	0.12 ± 0.02	0.08 ± 0.02
32.252	Selina-5,11-diene	-	0.07 ± 0.01
32.540	Humulene	0.27 ± 0.05	0.05 ± 0.01
32.882	Gamma-Selinene	-	0.23 ± 0.01
32.937	Alpha-Bergamotene	1.54 ± 0.20	0.17 ± 0.02
33.307	Valencene	-	0.81 ± 0.03
33.162	Germacrene D	6.44 ± 0.90	0.04 ± 0.01
33.312	Alpha-Farnesene	1.74 ± 0.19	-
33.401	Eremophilene	0.23 ± 0.03	-
33.405	Beta-Selinene	-	0.44 ± 0.02
33.462	Gamma-Muurolene	0.30 ± 0.03	-
33.517	Bicyclogermacrene	0.62 ± 0.08	0.41 ± 0.02
33.929	Delta-Cadiene	1.11 ± 0.12	-
34.161	Alpha-Maalliene	-	0.03 ± 0.01
34.326	Gamma-Cadinene	0.13 ± 0.02	0.18 ± 0.01
34.431	Alpha-Cadinene	0.12 ± 0.01	-
34.668	Gamma-Guaiene	-	0.03 ± 0.03
34.730	Nerolidol	1.57 ± 0.16	-
34.917	1(10),11-Eremophiladien-9-ol	-	0.04 ± 0.04
35.046	1,9-Aristoladiene	-	0.06 ± 0.06
35.493	Germacrene D-4-ol	1.24 ± 0.09	-
35.541	Beta-Spathulenol	-	0.11 ± 0.01
35.738	Caryophyllene oxide	0.18 ± 0.02	-
36.144	Beta-Oplopenone	0.16 ± 0.14	-
36.186	5-epi-7-epi-alpha-Eudesmol	-	0.17 ± 0.01
36.682	Epi-gamma-Eudesmol	-	1.13 ± 0.03
36.822	Eudesm-5-en-11-ol	-	2.78 ± 0.06
36.901	2-[(1aS,4aS,7R)-4a-methyldecahydrocyclopropa[d]naphthalen-7-yl]-2-propanol	-	0.29 ± 0.01
36.961	Tau-Cadinol	0.33 ± 0.02	-
37.014	Spirojatamol	0.59 ± 0.04	-
37.196	Beta-Gurjunene	-	0.06 ± 0.06
37.292	Alpha-Cadinol	0.89 ± 0.05	-
37.372	Cyclotridecane	0.33 ± 0.01	-
37.475	7-Methoxy-1H-indole-5-carboxylic acid	0.60 ± 0.08	-
37.491	1,2,5-Trimethylpyrrole	-	10.67 ± 0.36
37.680	Beta-Maaliene	2.15 ± 0.24	0.06 ± 0.01
37.696	Beta-Neoclovene	-	1.46 ± 0.03
37.870	(E)-3-(1-Phenylprop-1′-en-2′-yl))-pentane-2,4-dione	-	2.65 ± 0.48
38.141	Cyercene 1	-	0.28 ± 0.02
38.285	6,7-dimethoxy-3,4-dihydroisoquinoline	5.42 ± 0.06	-
38.508	(3E,5E,8Z)-3,7,11-trimethyldodeca-1,3,5,8,10 pentaene	-	0.53 ± 0.01
39.219	Eremophilone	-	0.21 ± 0.01
39.463	2,3-Dihydro-1H-cyclonona[def]biphenylene	-	0.59 ± 0.01
39.534	1-(2-Methoxyphenyl)-5-methyl-4-hexene-1-one	-	0.16 ± 0.001
39.606	n-Cetyl alcohol	1.54 ± 0.07	-
39.619	1-Hexadecanol	-	0.82 ± 0.02
40.630	1-Cyanoacetylpiperidine	-	1.08 ± 0.03
40.805	Neophytadiene	3.84 ± 0.43	-
40.920	3,7,11,15-tetramethylhexadec-2-ene	0.51 ± 0.06	-
40.964	Bakkenolid A	0.33 ± 0.02	3.75 ± 0.05
41.301	(Z)-1,3-Phytadiene	0.31 ± 0.27	
41.364	3-phenylbenzothieno[3,2-e]-1,2,4-triazine	-	0.09 ± 0.08
41.528	4-methylcyclohex-3-enecarbaldehyde	-	6.16 ± 0.38
41.737	Cetene	3.51 ± 0.24	1.97 ± 0.01
42.013	Cyclocolorenone	2.37 ± 0.20	-
42.112	1,2-Benzenediol, o-(3-cyclopentylpropionyl)-	-	0.53 ± 0.02
42.733	(3Z)-3a,4,5,6-Tetrahydro-1-hydroxy-3-(2-hydroxy-2-ethylbutylidene)azulen-2(1H)-one	-	1.21 ± 0.21
43.762	E-14-Hexadecenal	0.63 ± 0.01	-
43.784	1-Nonadecanol	-	0.60 ± 0.03
43.914	Ethyl palmitate	-	0.12 ± 0.001
44.221	5-Amino-8-cyano-7-methoxy-3,4-dihydro-3-methyl-1,6-naphthyridin-2(1H)-one	0.83 ± 0.05	-
44.386	3,4-dihydro-2-(methoxymethyl)-4,4-dimethyl-5-phenyl-2H-Pyran	-	30.03 ± 0.77
44.577	1-(7,8-dihydro-3-hydroxy-4-propyl-2-naphthalenyl)-Ethanone	-	0.72 ± 0.62
45.003	1-Octadecene	-	0.05 ± 0.05
45.622	Ligularenolide	-	0.11 ± 0.02
45.695	(5E)-5-Icosene	0.25 ± 0.22	-
45.762	Liguhodgsonal	0.46 ± 0.06	-
45.967	Alpha-Methyl linolenate	0.42 ± 0.13	-
45.993	Ligularone	-	2.00 ± 0.13
46.171	Phytol	11.51 ± 0.57	-
46.654	Drimenol	-	0.71 ± 0.62
46.718	Linoleic acid	2.08 ± 2.13	-
47.058	Ethyl linoleate	-	0.08 ± 0.01
48.269	13-Oxoellipticine	-	1.13 ± 0.08
52.944	2-Monopalmitin	1.50 ± 0.31	-
55.086	2-Methyl-5H-dibenzazepine	0.13 ± 0.11	-
55.692	1-Monolinolein	4.72 ± 0.66	-
55.829	2-Monolinolenin	4.04 ± 0.46	-
57.595	Squalene	3.75 ± 0.26	-
59.911	12-methoxy-18-norpodocarpa-8,11,13-trien-19-ol	-	5.05 ± 0.27
63.885	2-(5-methoxy-1-methyl-3-indolyl)acetic acid methyl ester	-	0.4 ± 0.02
64.502	Alpha-Tocopherol	0.40 ± 0.05	0.08 ± 0.07
65.932	Sesamin	1.79 ± 0.09	0.30 ± 0.02
68.779	Stigmasterol	5.89 ± 0.02	-
70.968	Gamma-Sitosterol	6.10 ± 0.39	-
72.957	Beta-Amyrin	1.11 ± 0.06	-
81.239	1,2-Benzenediol, 4-(1-methyl-4-piperidinyl)-	-	0.36 ± 0.31
84.315	4,5,6,7-tetraphenyl-1H-inden-1-one	-	0.74 ± 0.04

## Data Availability

Not applicable.

## Supplementary Materials

The following supporting information can be downloaded at: https://www.mdpi.com/article/10.3390/plants11213005/s1, Figure S1: GC-MS chromatography of leaf and root extracts from *L. fischeri*. Figure S2: Principal component analysis (PCA) score plot of the non-polar compounds in roots and leaves of *L. fischeri*.
