# Supplementary material for: Phytochemical Composition and Antioxidant and Anti-Inflammatory Activities of Ligularia fischeri Turcz: A Comparison between Leaf and Root Extracts
Source: Plants (Basel). 2022 Nov 7;11(21):3005. doi: 10.3390/plants11213005 (PMC9656575; doi:10.3390/plants11213005)
Supplement: Supplementary file 1 [file plants-11-03005-s001.zip › plants-1972991-supplementary.pdf]

Supplementary Material

Phytochemical Composition and Antioxidant and Anti-Inflammatory Activities of *Ligularia fischeri* Turcz: A comparison between Leaf and Root Extracts

(A)

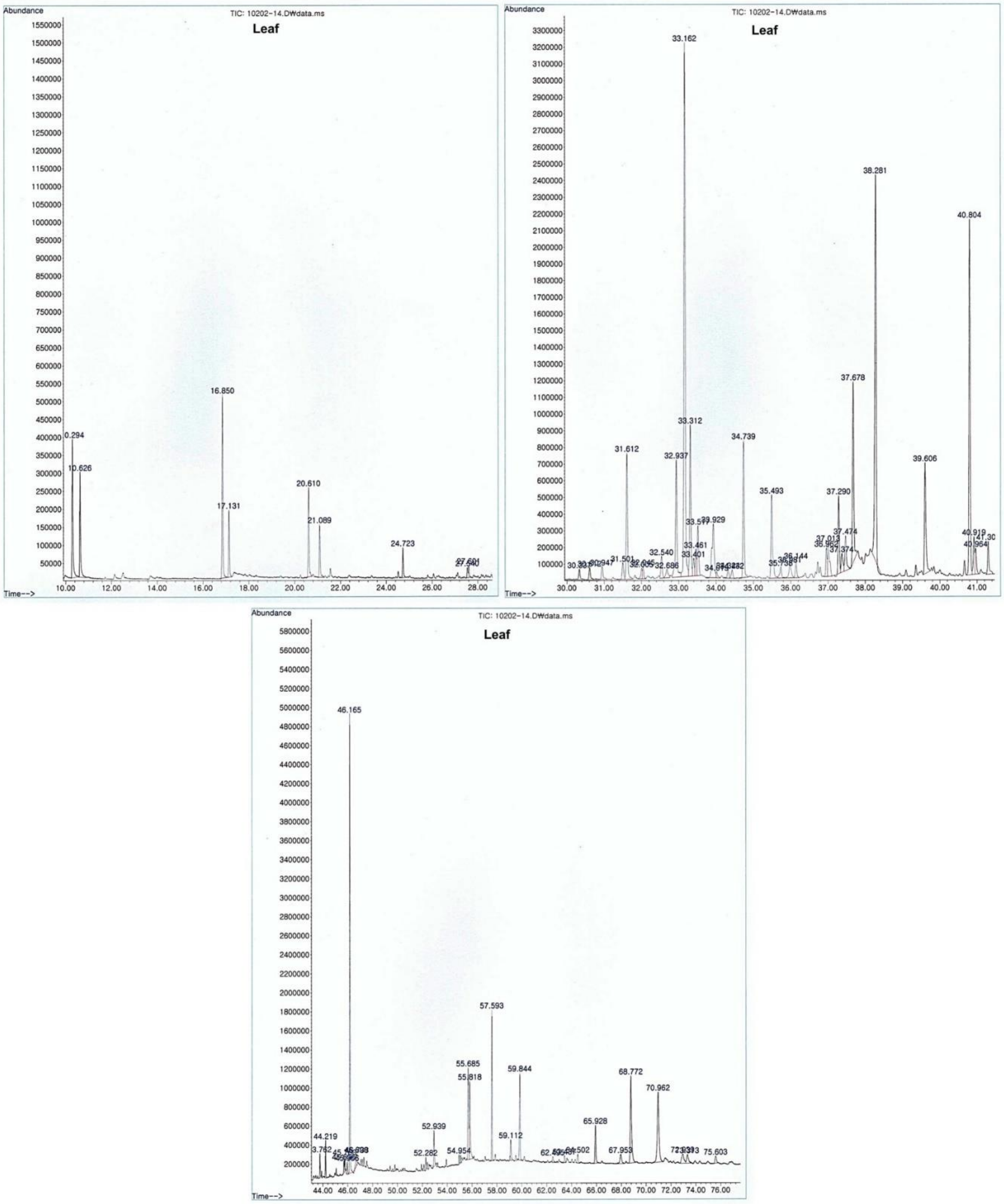

(B)

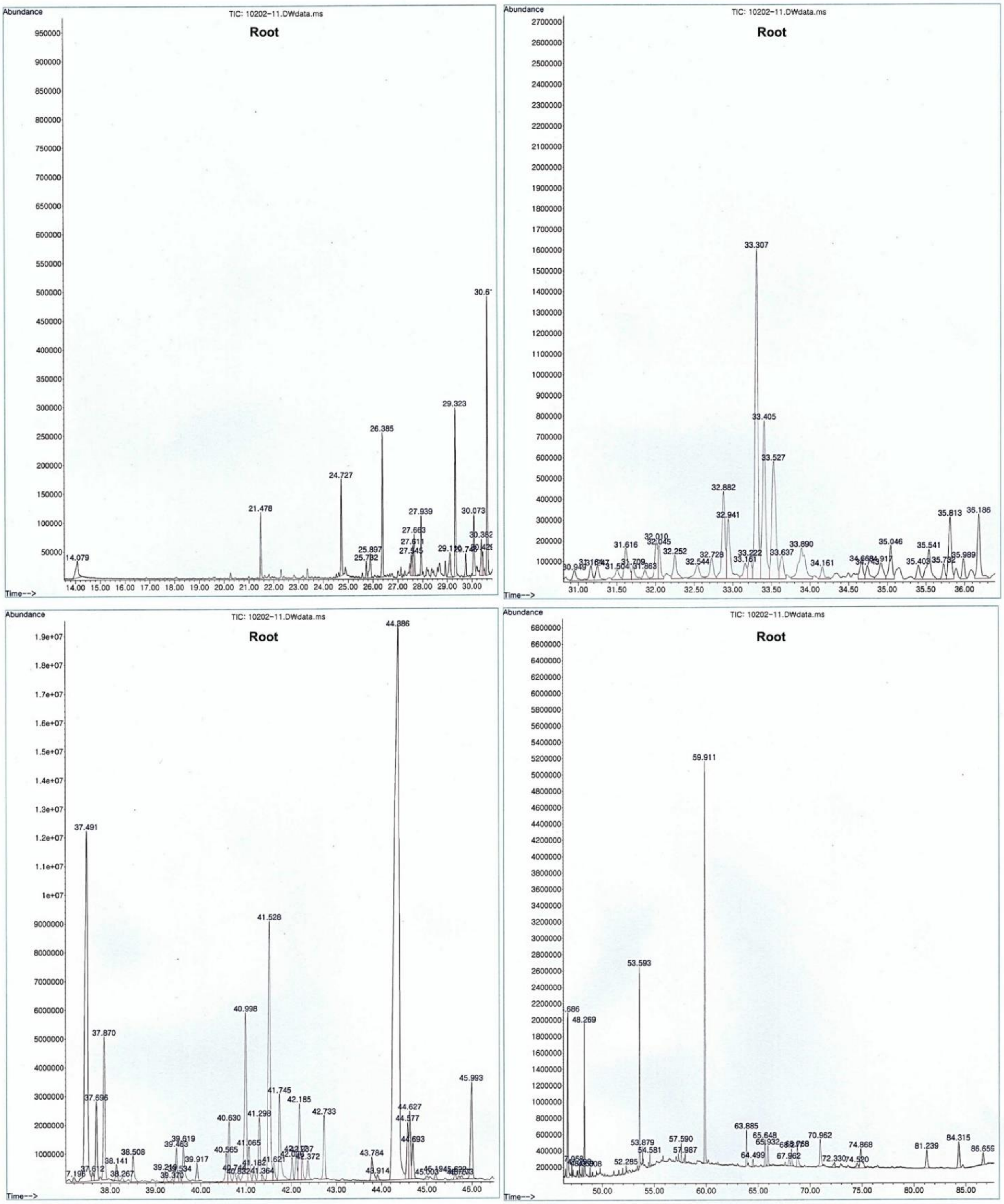

Figure S1. GC chromatograms of (A) leaf and (B) root extracts from *L. fischeri*.

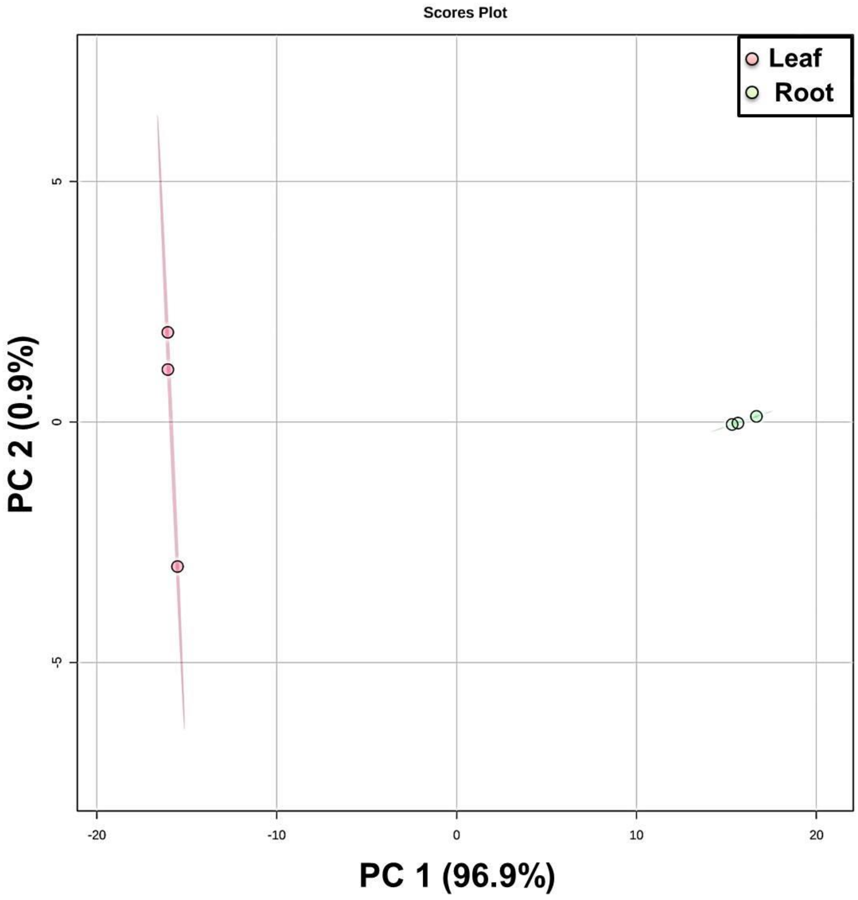

Figure S2. Principal component analysis (PCA) score plot of the non-polar compounds in roots and leaves of *L. fischeri*. Red dots represent non-polar compounds in leaves. Green dots represent non-polar compounds in roots.
